# Supplementary material for: Time‑resolved multi-omic analysis of paclitaxel exposure in human iPSC‑derived sensory neurons unveils mechanisms of chemotherapy‑induced peripheral neuropathy
Source: Cell Death Dis. 2026 Feb 10;17(1):211. doi: 10.1038/s41419-026-08445-2 (PMC12921266; doi:10.1038/s41419-026-08445-2)
Supplement: Supplementary file 15 — Dataset 1. [file 41419_2026_8445_MOESM15_ESM.zip › CDD_Open_Data/04_RNA_Seq/Timeline_panel_plots_etc_2023-01-23_1609.zip!/Panel_Plots_etc/08_tmod_panel_plot_msigdb_kegg_pval_filter_0.05_pval0.05_AUC0.5_L2FC0.5.pdf]

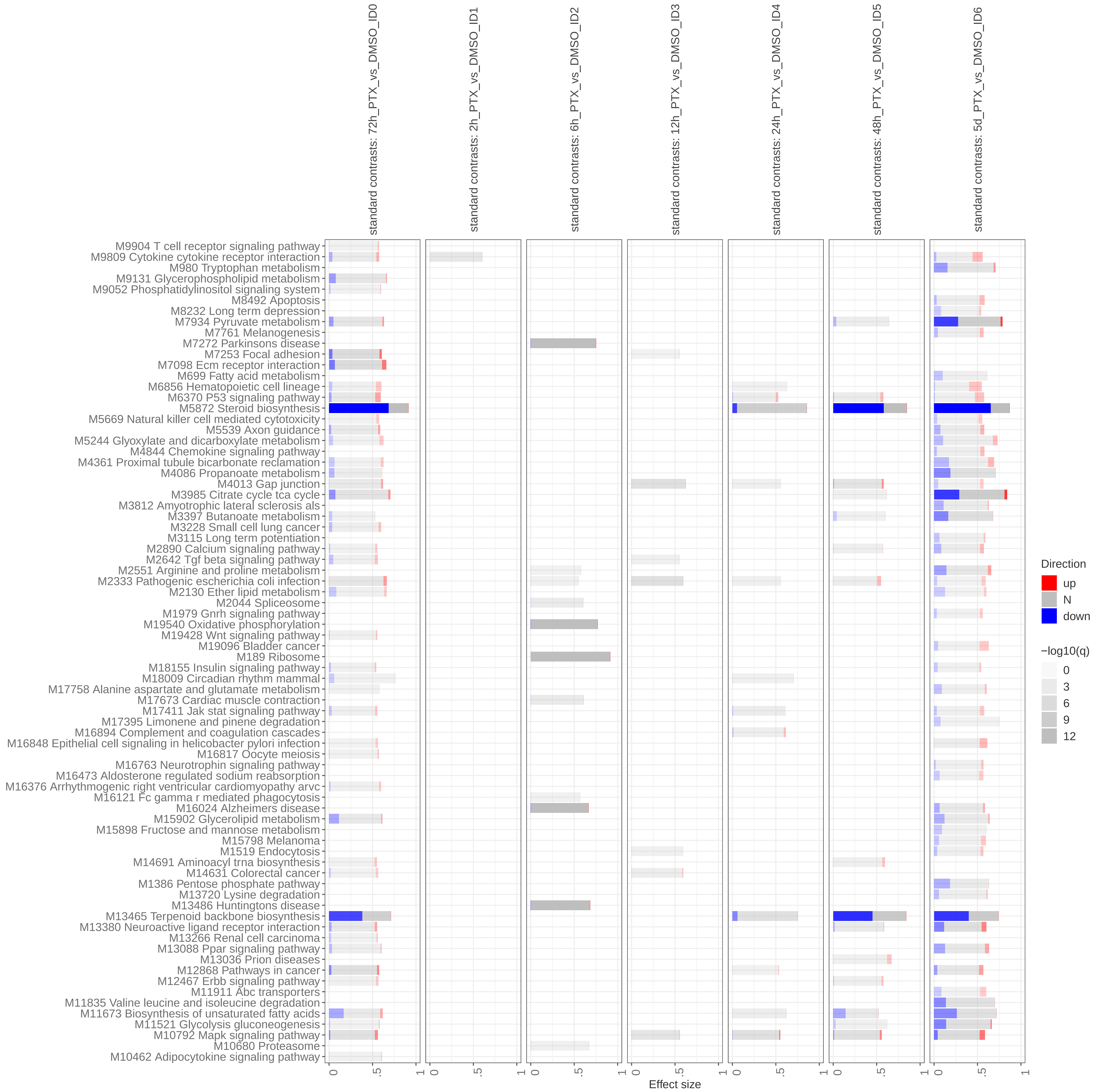
standard contrasts: 72h_PTX_vs_DMSO_ID0
standard contrasts: 2h_PTX_vs_DMSO_ID1
standard contrasts: 6h_PTX_vs_DMSO_ID2
standard contrasts: 12h_PTX_vs_DMSO_ID3
standard contrasts: 24h_PTX_vs_DMSO_ID4
standard contrasts: 48h_PTX_vs_DMSO_ID5
standard contrasts: 5d_PTX_vs_DMSO_ID6
M9904 T cell receptor signaling pathway
M9809 Cytokine cytokine receptor interaction
M980 Tryptophan metabolism
M9131 Glycerophospholipid metabolism
M9052 Phosphatidylinositol signaling system
M8492 Apoptosis
M8232 Long term depression
M7934 Pyruvate metabolism
M7761 Melanogenesis
M7272 Parkinsons disease
M7253 Focal adhesion
M7098 Ecm receptor interaction
M699 Fatty acid metabolism
M6856 Hematopoietic cell lineage
M6370 P53 signaling pathway
M5872 Steroid biosynthesis
M5669 Natural killer cell mediated cytotoxicity
M5539 Axon guidance
M5244 Glyoxylate and dicarboxylate metabolism
M4844 Chemokine signaling pathway
M4361 Proximal tubule bicarbonate reclamation
M4086 Propanoate metabolism
M4013 Gap junction
M3985 Citrate cycle tca cycle
M3812 Amyotrophic lateral sclerosis als
M3397 Butanoate metabolism
M3228 Small cell lung cancer
M3115 Long term potentiation
M2890 Calcium signaling pathway
M2642 Tgf beta signaling pathway
M2551 Arginine and proline metabolism
M2333 Pathogenic escherichia coli infection
M2130 Ether lipid metabolism
M2044 Spliceosome
M1979 Gnrh signaling pathway
M19540 Oxidative phosphorylation
M19428 Wnt signaling pathway
M19096 Bladder cancer
M189 Ribosome
M18155 Insulin signaling pathway
M18009 Circadian rhythm mammal
M17758 Alanine aspartate and glutamate metabolism
M17673 Cardiac muscle contraction
M17411 Jak stat signaling pathway
M17395 Limonene and pinene degradation
M16894 Complement and coagulation cascades
M16848 Epithelial cell signaling in helicobacter pylori infection
M16817 Oocyte meiosis
M16763 Neurotrophin signaling pathway
M16473 Aldosterone regulated sodium reabsorption
M16376 Arrhythmogenic right ventricular cardiomyopathy arvc
M16121 Fc gamma r mediated phagocytosis
M16024 Alzheimers disease
M15902 Glycerolipid metabolism
M15898 Fructose and mannose metabolism
M15798 Melanoma
M1519 Endocytosis
M14691 Aminoacyl trna biosynthesis
M14631 Colorectal cancer
M1386 Pentose phosphate pathway
M13720 Lysine degradation
M13486 Huntingtons disease
M13465 Terpenoid backbone biosynthesis
M13380 Neuroactive ligand receptor interaction
M13266 Renal cell carcinoma
M13088 Ppar signaling pathway
M13036 Prion diseases
M12868 Pathways in cancer
M12467 Erbb signaling pathway
M11911 Abc transporters
M11835 Valine leucine and isoleucine degradation
M11673 Biosynthesis of unsaturated fatty acids
M11521 Glycolysis gluconeogenesis
M10792 Mapk signaling pathway
M10680 Proteasome
M10462 Adipocytokine signaling pathway
0 .5 1
Effect size
Direction
up
N
down
−log10(q)
0
3
6
9
12
